# Lipid production from lignocellulosic biomass using an engineered *Yarrowia lipolytica* strain

**DOI:** 10.1186/s12934-022-01951-w

**Published:** 2022-10-28

**Authors:** Katarzyna Drzymała-Kapinos, Aleksandra M. Mirończuk, Adam Dobrowolski

**Affiliations:** 1 Department of Biotechnology and Food Microbiology, Wrocław University of Environmental and Life Sciences, 37 Chełmońskiego Street, 51-630 Wrocław, Poland; 2 Present Address: Laboratory for Biosustainability, Institute of Environmental Biology, Wrocław University of Environmental and Life Sciences, Wrocław, Poland

**Keywords:** *Yarrowia lipolytica*, Lignocellulosic wastes, Xylose utilization, Microbial lipids

## Abstract

**Background:**

The utilization of industrial wastes as feedstock in microbial-based processes is a one of the high-potential approach for the development of sustainable, environmentally beneficial and valuable bioproduction, inter alia, lipids. Rye straw hydrolysate, a possible renewable carbon source for bioconversion, contains a large amount of xylose, inaccessible to the wild-type *Yarrowia lipolytica* strains. Although these oleaginous yeasts possesses all crucial genes for xylose utilization, it is necessary to induce their metabolic pathway for efficient growth on xylose and mixed sugars from agricultural wastes. Either way, biotechnological production of single cell oils (SCO) from lignocellulosic hydrolysate requires yeast genome modification or adaptation to a suboptimal environment.

**Results:**

The presented *Y. lipolytica* strain was developed using minimal genome modification—overexpression of endogenous xylitol dehydrogenase (XDH) and xylulose kinase (XK) genes was sufficient to allow yeast to grow on xylose as a sole carbon source. Diacylglycerol acyltransferase (DGA1) expression remained stable and provided lipid overproduction. Obtained an engineered *Y.* *lipolytica* strain produced 5.51 g/L biomass and 2.19 g/L lipids from nitrogen-supplemented rye straw hydrolysate, which represents an increase of 64% and an almost 10 times higher level, respectively, compared to the wild type (WT) strain. Glucose and xylose were depleted after 120 h of fermentation. No increase in byproducts such as xylitol was observed.

**Conclusions:**

Xylose-rich rye straw hydrolysate was exploited efficiently for the benefit of production of lipids. This study indicates that it is possible to fine-tune a newly strain with as minimally genetic changes as possible by adjusting to an unfavorable environment, thus limiting multi-level genome modification. It is documented here the use of *Y. lipolytica* as a microbial cell factory for lipid synthesis from rye straw hydrolysate as a low-cost feedstock.

**Supplementary Information:**

The online version contains supplementary material available at 10.1186/s12934-022-01951-w.

## Background

The development of environmentally friendly branches of the biofuel industry is indispensable to establishing the definite suitability of the biorefinery concept. Microbial oils, in contrast to the traditionally used plant oils, have the potential to partially reduce the use of food raw materials for energy purposes and reduce the potential risk of deforestation to acquire new arable land. Additionally, in the process of developing the single cell oils (SCO) production, it is possible to plan targeted changes of the fatty acid profile [1–3]. Therefore, many attempts have been made to create a biological factory based on the microbial cells. The lipid production should take a twin-track approach. The first issue is the selection of inexpensive carbon sources as alternatives to the commonly used glucose; thus, attention should be paid to the opportunity of reuse of waste materials obtained from various branches of industry. Some of the previous research has shown that used cooking oil [4, 5], hydrolyzed castor oil [6], olive-mill wastewater [7, 8], crude glycerol [1, 9–12], molasses [13, 14] or food waste hydrolysates [15, 16] could be useful as feedstock for yeast bioprocesses. Lignocellulosic material, which can be hydrolyzed and converted to monosaccharides, appears to be another good candidate for low-cost feedstock [17–20]. Three varied fractions, cellulose, hemicellulose and lignin, have been identified from lignocellulosic biomass. Due to the chemical composition of plant cell walls, lignocellulosic hydrolysate is mainly a mixture of glucose and xylose. The high content of xylose, which is poorly metabolized by the most microorganism, due to the carbon catabolite repression suppressing the assimilation of C5 sugars, is one of the obstacles limiting the applications of lignocellulose [21]. In the present study, rye straw, which is a commonly used non-wood waste agricultural raw material in European countries, was used as a potential carbon source. The compositional analysis of the rye straw hydrolysate revealed a diverse sugar content—over 60% xylose, 27% glucose and 9% arabinose, in total 28 g/L monosaccharaides [22]. As a consequence, the second issue is choice of the most appropriate microbial factory cell. Rye straw hydrolysate has already been used for ethanol fermentation by *Saccharomyces cerevisiae*. However, xylose was detected in hydrolysate after the process [23].

In the context of biofuel production, *Y. lipolytica* appears justified as a microbe of interest. *Y. lipolytica* is a non-conventional, but well-studied, oleaginous yeast with huge industrial potential. In recent years, *Y. lipolytica* has been widely used for biosynthesis of metabolites such as citric acid [10, 24], polyols [25], enzymes [26], terpenoids [27–29] and polyketides [30, 31]. The high capacity of *Y. lipolytica* to convert carbon source into lipid provides an attractive platform for single cell oil (SCO) production. Lipid metabolism in this yeast has been expanded considerably. The overexpression of endogenous genes related to the lipid synthesis pathway caused a well-documented increased amount of SCO [1]. Tai and Stephanopoulos proposed compact strategy co-expression of *ACC1* and *DGA1* [32]. Furthermore, lipid production can be enhanced by deletion of genes related to lipid degradation such as *MFE2* or genes associated with peroxisome biogenesis [33–35]. Additionally, evolution-based strategies were used as a tool for improving lipid accumulation [36]. The combination of increased lipid accumulation with efficient growth on a lignocellulosic substrate could lead to the creation of a biotechnological chassis for viable lipid production. One obstacle is that commonly used laboratory *Y. lipolytica* strains for biotechnology applications are unable to utilize xylose as its sole carbon source. The xylose utilization pathway, apart from the necessary transporters, requires activity of xylose reductase (XR), which mediates conversion of xylose to xylitol, xylulose dehydrogenase (XDH), which converts xylitol to xylulose, and xylulose kinase (XK) for final conversion to xylulose-5-phospate. Xylose-degrading enzymes have been confirmed in the *Y. lipolytica* system [37], nevertheless, without adaptation or genetic modification, yeast growth is insufficient. Table 1 summarizes research to date on the use of xylose utilization or co-fermentation of mixed sugars. The fermentation based on pure xylose should be considered as preliminary research for further use of xylose-rich wastes or as basic and necessarily metabolic studies.

**Table 1:** Comparison of xylose and waste-based fermentation of previously reported *Y. lipolytica*

*Yarrowia lipolytica* strain	Substrate	Products			Source
		Biomass	Lipids	Others	
Y14: ATCC201249 Δ*Ku70 ssXR ssXDH ylXK DS PPDS ATR1 tHMG1ERG9 ERG20 TAL TKL TX* (xylose-adaptive strain)	Xylose (batch fermentation)	OD_600_ 39.01	–	PPD 98.23 mg/L	[38]
YSXID: PO1f *Δpex10::DGA1 ylXK XylA* (xylose-adaptive strain)	Miscanthus hydrolysate (fed-batch fermentation supplemented with pure sugar)	28.32 g/L^a^	12.01 g/L	NM	[39]
Po1t	Xylose (batch co-fermentation with crude glycerol)	OD_600_ 31.8	–	xylitol 50.5 g/L	[40]
ALA-A x XUS-B *L36DGA1 2xRkD12-15 MATA1 MATA2 x* E26 *4xXYL1 4xXYL2 ylXKS MATB1 MATB2*	Xylose (batch fermentation)	15 g/L	6 g/L	NM	[41]
Po1f *ylXDH* + *ylXKS*	Xylose	1 g/L	0.21 g/L	NM	[37]
YlSR102 PO1f ylXDH (xylose-adaptive strain)	Xylose	OD_600_ 7.39	NM	xylitol ca.1 g/L	[42]
E26 XUS E26 *ssXR ssXDH*	Xylose (batch fermentation)	OD_600_ ca. 120	15 g/L	NM	[43]
XYL + Obese PO1d *Δpox1-6 Δtgl4 GPD1 DGA2 ssXR ssXDH ylXK*	Xylose	12.98 g/L	6.16 g/L	citric acid 3.46 g/L xylitol 1.29 g/L	[44]
	Xylose (batch co-fermentation with glycerol)	120 g/L	50.5 g/L	Citric acid 1.0 g/L Xylitol 11.1 g/L	
PSA02004PP PSA02004 *ylXK ylXDH ylXR*	xylose-rich hydrolysate derived from sugarcane bagasse (batch fermentation)	5.3 g/L	–	Succinic acid 5.6 g/L Acetic acid 8.5 g/L	[45]
ylXYL + Obese-XA PO1d *Δpox1-6 Δtgl4 GPD1 DGA2 ylXR ylXDH ylXK XPKA ACK*	agave bagasse hydrolysate	25.8 g/L	16.5 g/L	–	[46]

*PPD* protopanaxadiol, *NM* not mentioned, *ss* gene derived from *Schefferomyces stipitis*, *yl* gene derived from *Y. lipolytica*

^a^Calculated based on paper

The purpose of this study was to examine the feasibility of using rye straw hydrolysate as a carbon source for the engineered *Y. lipolytica* strain. The modified strain, with limited genome modification, using exclusively native genes, was able to convert the entire sugar pool from low-cost lignocellulosic hydrolysate and produce a significant amount of lipids.

## Results

### Overexpression of the endogenous xylose utilization pathway and improving growth kinetics

**Fig. 1:**
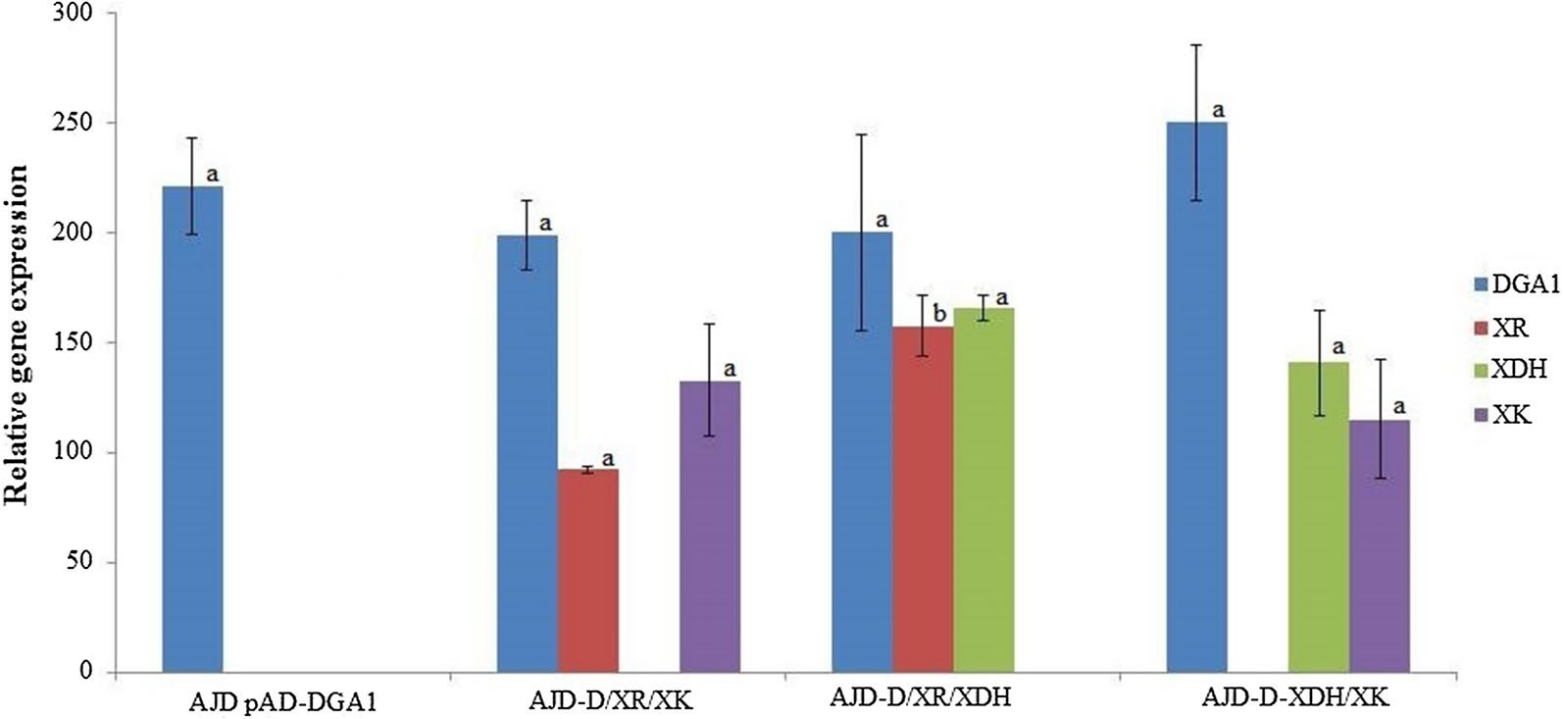
Relative quantification of RNA transcript in the engineered strains. The histograms show the relative expression of genes with respect to the control—actin was used as a reference gene and *Y. lipolytica* A101 was used as a control strain. Samples were analyzed in triplicate. One-way ANOVA at p ≤ 0.05 was calculated. The homogeneous groups between the same genes according to the Duncan test were estimated and they are represented by the same letter

The wild-type *Y. lipolytica* A101 strain is incapable of growing on xylose as a sole carbon source, which was confirmed in the presented studies and which is quite common for biotechnological relevant strains [44, 47]. The aim of the study was to develop a new strain with as few genetic changes as possible. Endogenous xylose reductase, xylitol dehydrogenase and xylulose kinase were integrated in pairs into an AJD pAD-DGA1 strain [48]. The parental yeast strain with overexpression of *DGA1* (YALI0E32769g) is characterized by increased lipid production and accumulation [1, 49]. Strains were prepared according to the scheme in Additional file 1: Fig. S1. To achieve high protein expression, all genes were overexpressed under the control of the UAS1_B16_-TEF hybrid promoter [50]. The expression level of the functional genes was evaluated by qRT-PCR. The results showed that the engineered strains exhibit high levels of gene expression (Fig. 1). Lack of statistically different magnitude of relative gene expression, provided by the high-strength promoter, allows the comparison of the obtained strains under identical growth-medium conditions. The expression level of the previously integrated *DGA1* gene also remained at a similar level in all tested strains.

**Fig. 2:**
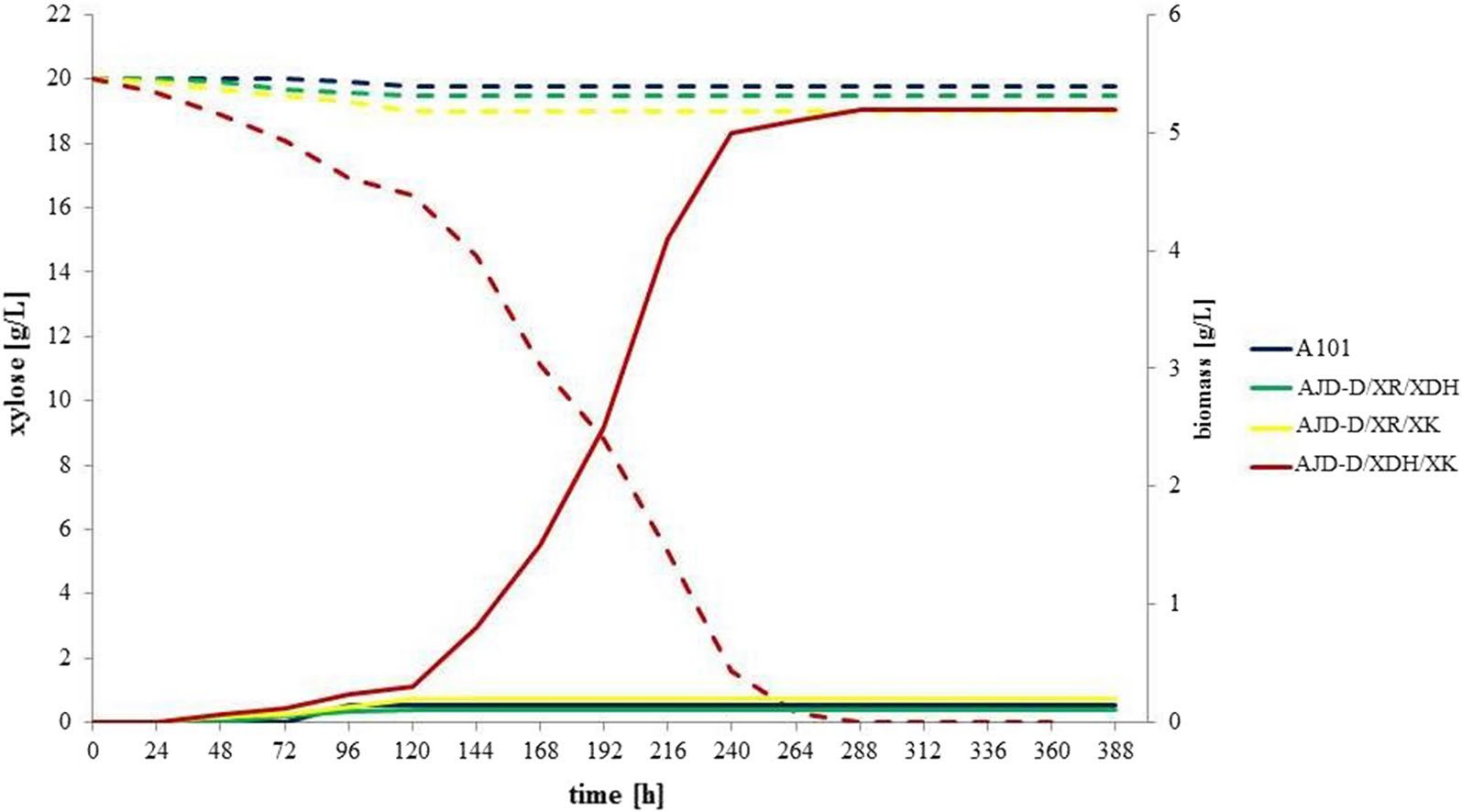
Growth of different *Y. lipolytica* engineered strains on xylose—(I) biomass increase (solid line) of *Y. lipolytica* A101, AJD-D/XR/XK (introducing heterologous xylose reductase and xylulose kinase), AJD-D/XR/XDH (introducing heterologous xylose reductase and xylitol dehydrogenase) and AJD-D/XDH/XK (introducing heterologous xylitol dehydrogenase and xylulose kinase), (II) xylose concentration (dotted line)

Next, the engineered strains were tested for growth ability under sole xylose conditions in a shake-flask experiment. After 120 h AJD-D/XDH/XK began the increase of biomass exclusively. In Fig. 2 the growth and xylose assimilation curves are presented. At the end of the 12-day period, the biomass of AJD-D/XDH/XK reached the level of 5.2 g/L (the biomass total productivity Q_x_ = 18.06 ± 1.13 mg/L/h, the biomass total yield Y_x_ = 0.26 g/g). The remaining strains did not demonstrate a sufficient capacity for growth.

**Fig. 3:**
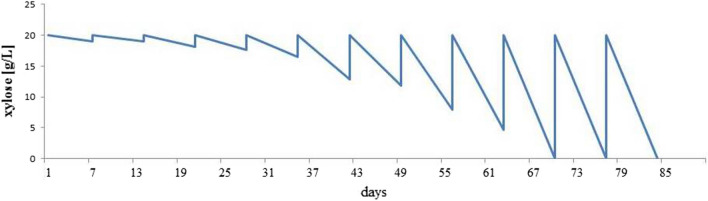
Changes in xylose assimilation from the medium during culture AJD-D/XDH/XK strain, performed by sequential serial passages in shake cultural tube. At intervals an aliquot of the culture was transferred to a new medium in the tube. Each medium contained 20 g/L xylose supplemented with 6.7 g/L YNB. The strain after the last passages was used in the next research and has been described as *Y.* *lipolytica* AJD-D/XYL/ALE

It was assumed that prolonged exposure to an unfavorable, but cell-growth-enabling medium composition, would lead to changes *Y. lipolytica* gene expression level and, consequently, a change in phenotype [51]. Despite obtaining a strain able to grow on pure xylose, the long duration of the lag phase was a predicament. Instead of introducing further genetic modifications, it was decided to use time-extended shake-flask culture to accelerate cell growth. The starting point was the YNB medium consisting of 20 g/L xylose inoculated with starved yeast cells. The starved strain was previously cultured on YNB medium supplemented with glucose and left under carbon deficient conditions for a 10 days, then double-washed with sterile water and transferred to appropriate xylose-based medium. The yeast was transferred to fresh medium at an interval of 7 days. Changes in xylose assimilation between successive passages are shown in Fig. 3. In the early passages the sugar concentration decreased slightly. With each subsequent passage, the yeast began to utilize xylose more and more efficiently. Finally obtained yeast cell population with the ability to assimilate 20 g/L of xylose within 7 days was considered homogeneous and was named AJD-D/XYL/ALE.

**Fig. 4:**
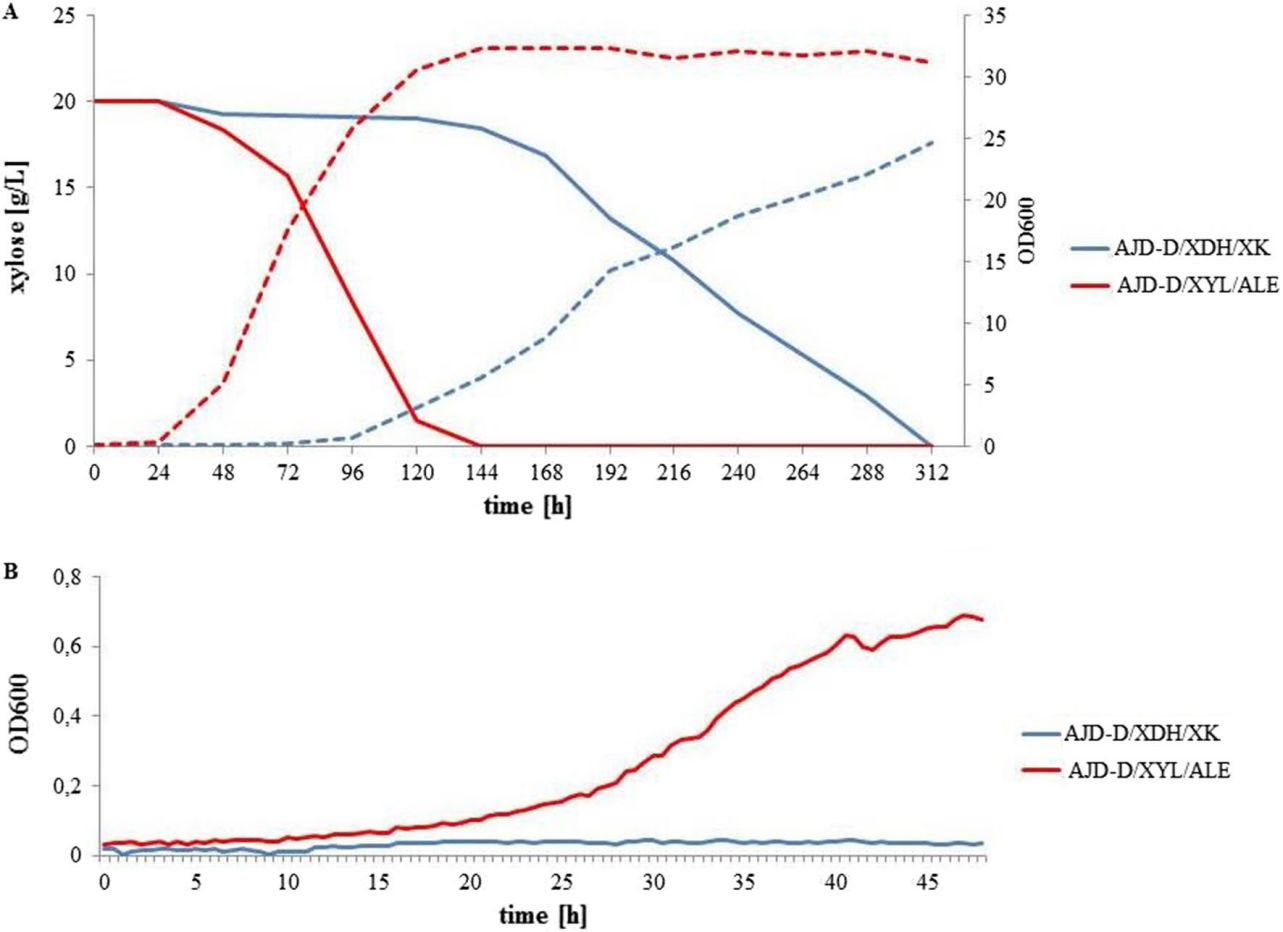
Variations in growth characteristics between wild type, AJD-D/XDH/XK and AJD-D/XYL/ALE. (A) OD600 (dashed line) and xylose concentration (solid line) curves. (B) OD600 during first 45 h of growth on media containing 20 g/L xylose supplemented with 6.7 g/L YNB

Differences in growth kinetics between parental and newly developed strains were examined in a shake-flask experiment. The OD600 and xylose assimilation curves are presented in Fig. 4A. The strain AJD-D/XDH/XK started to grow in the logarithmic phase after 96 h. The overall xylose assimilation occurred after 312 h, and the final optical density reached a level less than 25. The strain AJD-D/XYL/ALE utilized the total amount of xylose during 144 h and attained the highest grown parameters, such as xylose consumption rate 138.89 ± 3.37 mg/L/h and optical density level—OD600 = 32.3. In comparison, the initial strain after 144 h OD600 stood at 5.6 and xylose consumption rate achieved 11.11 ± 0.41 mg/L/h. The duration of the lag phase was significantly reduced, which was observed through the microplate reader (Fig. 4B).

### Lipid production from waste-based medium

**Fig. 5:**
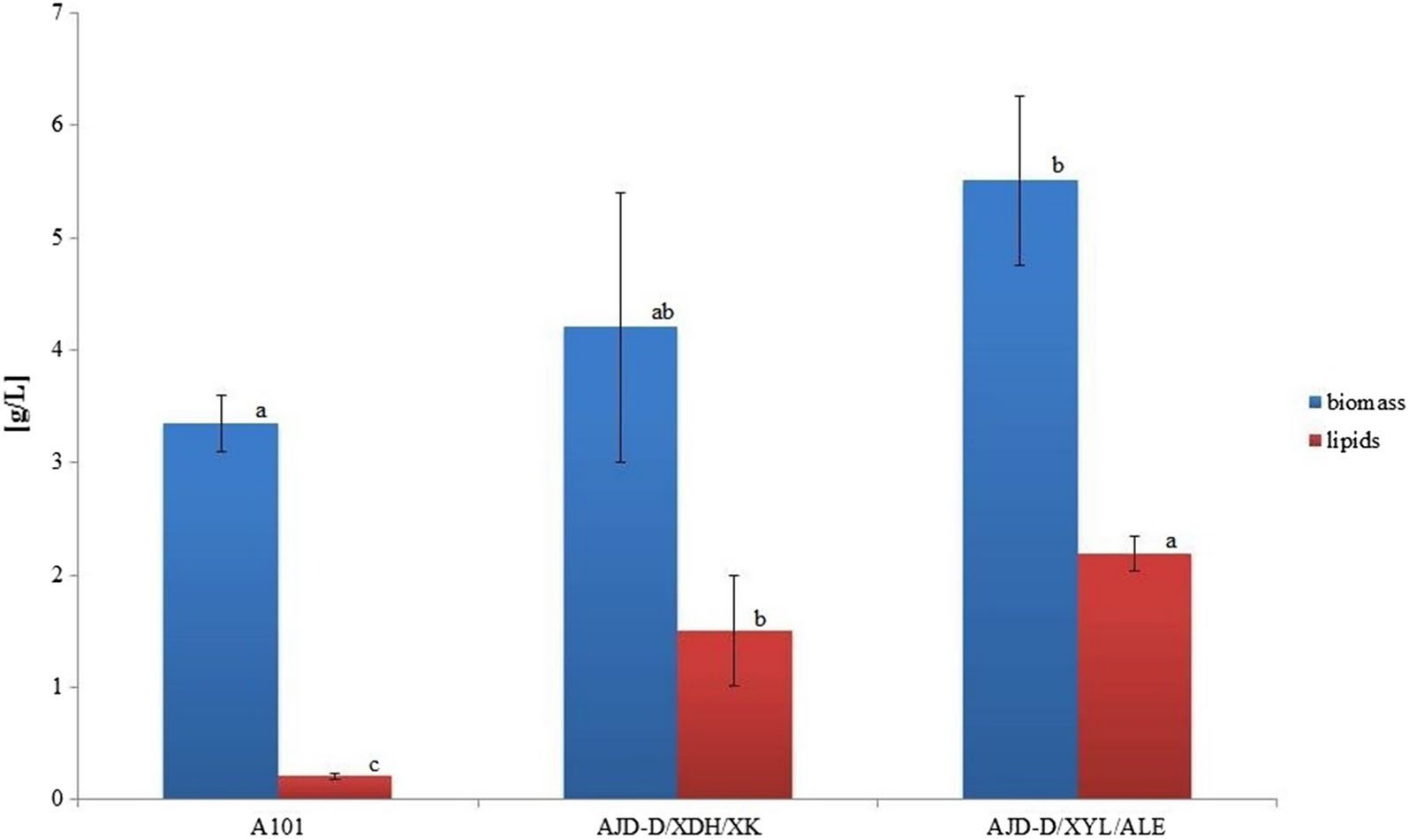
Parameters of biomass and lipid production by *Y. lipolytica* A101, AJD-D/XDH/XK and AJD-D/XYL/ALE grown on rye straw hydrolysates containing about 61% xylose, 27% glucose, 8% arabinose and insignificant amounts of mannose or galactose, supplemented with YNB. Data from triplicate fermentations are shown. One-way ANOVA at p ≤ 0.05 was calculated and homogeneous groups were estimated according to the Duncan test. Mean values over the bars that are not significantly different from each other (p > 0.05) are represented by the same letter

Rye straw is one of the many lignocellulosic agricultural wastes that can play a prominent role in replacing pure sugars, such as glucose, as a primary substrate used in the biotechnological industry. The main sugar found in straw hydrolysate is xylose, over 61%. The remainder of the sugar composition consists of glucose and arabinose [22]. To explore the feasibility of rye straw hydrolysate fermentation by the newly obtained strain of *Y. lipolytica* a shake-flask experiment with the new substrate was performed. Co-expression of the xylose metabolism pathway with diacylglycerol acyltransferase (DGA1) should ensure biovalorization of the waste substrate into SCO. After 120 h, the parental strain *Y. lipolytica* A101 reached only 3.35 g/L of biomass and 0.2 g/L of triacylglycerols (Fig. 5). The strain AJD-D/XYL/ALE greatly increased the biomass level by more than 60% and lipid level by almost tenfold at 5.51 g/L and 2.19 g/L, respectively. All sugars in the medium were depleted. Importantly, no xylitol or citric acid production was observed during the fermentation process. The total yield and productivity are shown in Table 2.

**Table 2:** Lipids and biomass production by *Y. lipolytica* strains grown on rye straw hydrolysates supplemented with YNB at the end of the shake-flask experiment

Strain	Biomass (g/L)	Yc x/s (g/g)	Qx (g/L·h)	TAG (g/L)	TAG (%)	Yc l/s (g/g)	Ql (g/L·h)
A101	3.35 ± 0.25	0.12 ± 0.01	0.03 ± 0.01	0.20 ± 0.03	6.01 ± 0.40	0.08 ± 0.01	0.002 ± 0.01
AJD-D/XDH/XK	4.20 ± 1.20	0.15 ± 0.04	0.04 ± 0.01	1.50 ± 0.50	35.68 ± 2.07	0.05 ± 0.02	0.015 ± 0.05
AJD-D/XYL/ALE	5.51 ± 0.75	0.20 ± 0.01	0.05 ± 0.01	2.20 ± 0.15	36.70 ± 1.05	0.07 ± 0.05	0.02 ± 0.01

Data from triplicate fermentations are shown

Yc x/s: the biomass total yield; Qx: the biomass total productivity; Yc l/s: the lipid total yield; Ql: the lipid total productivity

The fatty acid composition of the obtained biomasses was analyzed and is shown compared to rapeseed oil in Table 3. Overexpression of *DGA1* not only increases the lipid accumulation but also affects the change of lipid profile. Particularly high levels of oleic acid (C18:1) were observed in the modified strains with a simultaneously reduced amount of linoleic acid (C18:2). Oleic acid accounted for more than 63% of all fatty acid in the lipid pool in the AJD-D/XYL/ALE biomass. Interestingly, long-chain docosanoic acid, C22:0, and tetracosanoic acid, C24:0, were identified in the yeast biomass, with the highest amount detected in the biomass of the strain AJD-D/XYL/ALE.

**Table 3:** Percent of total cellular lipids of *Y. lipolytica* biomass obtained on hydrolysates-based medium supplemented by YNB compared to rapeseed oil fatty acid profile

Fatty acid	A101	AJD-D/XDH/XK	AJD-D/XYL/ALE	Rapeseed oil^a^
16:0	7.75 ± 0.02	9.60 ± 0.01	10.46 ± 1.19	3.50
16:1	8.65 ± 0.02	7.35 ± 0.02	5.01 ± 0.34	0.00
17:1	0.49 ± 0.21	0.43 ± 0.01	0.58 ± 0.32	0.00
18:0	3.26 ± 0.10	8.88 ± 0.02	10.64 ± 2.36	0.90
18:1	50.94 ± 0.18	58.87 ± 0.42	63.82 ± 2.77	64.40
18:2	27.51 ± 0.15	11.28 ± 0.03	6.95 ± 0.60	22.30
18:3	0.00	0.00	0.00	8.20
22:0	0.00	0.25 ± 0.01	0.43 ± 0.05	0.00
24:0	0.45 ± 0.20	1.26 ± 0.02	1.48 ± 0.22	0.00
SFA	11.46	19.99	23.01	4.40
MUFA	60.08	66.65	69.41	64.40
PUFA	27.51	11.28	6.96	30.50
Total	99.05	97.92	99.37	99.30
Others	0.95	2.08	0.63	0.70

Values are means ± SD of three determinations

^a^Based on [53]

## Discussion

The effective production of valuable metabolites is closely related to the complete utilization of the carbon source in the medium. Lignocellulosic waste materials, such as straw, may provide varied sugar composition by introducing glucose, xylose, or arabinose, among others, into the medium. Previous studies have shown that acid-enzymatic hydrolysis of rye straw allows one to obtain a sugar blend available for yeast growth [22]. However, the amount of yeast biomass produced was at a moderate level. The preliminary view was taken that improving xylose assimilation appeared essential to increase the effectiveness of biomass and lipid production from lignocellulosic materials. Therefore, a number of researchers have attempted to modify *Y. lipolytica* to utilize xylose. Initial attempts to engineer xylose metabolism by the heterologous expression of *Scheffersomyces stipitis* genes, a microbial model for xylose metabolism, failed to achieve strain able to grown on xylose, until after overexpressed three genes involved in xylose utilization pathway and received strain efficiently grown on xylose but simultaneously produced xylitol [44]. Similar growth parameters were achieved by the overexpression of the native genes [46]. Different approach in which introduced mutated xylose isomerase gene and performed adaptive laboratory evolution also resulted in receiving new *Y. lipolytica* strain enable to utilize xylose [39]. The aim of the present study was to reduce the amount of genetic modification and as has already been pointed out, that the double overexpression of xylitol dehydrogenase (XDH) and xylulose kinase (XK) is sufficient to enable *Y. lipolytica* to grow on xylose as the sole carbon source without adaptation [37]. However, the long lag phase is a bottleneck during production processes. Appropriate tool for metabolic changes, avoiding additional genetic modification, is adaptive laboratory evolution—a process based on natural evolution and selection directed to obtaining a better fitness to adapt to specific environmental conditions. ALE has been employed for an improved various important parameters in different aspects of biotechnology [52]. ALE was also proved to be effective for a wild-type *Y. lipolytica* strain and after adaptation yeast grew on xylose, but the growth rate was negligible. It has also been shown that strong expression of xylitol dehydrogenase is considered a priority factor for xylose metabolism [42]. Although ALE was not applied in the full sense in the present study, the simpler technique of prolonged culture in suboptimal medium already achieved a reduction in the time to reach the logarithmic phase of growth, which was an essential point in the creation of the new strain. Potential changes in genotype and phenotype stability remained unstudied, which would require genome sequencing of the adopted strain.

Therefore, the UAS1B_16_-TEF promoter, which provides high levels of gene expression, was used to construct the strain. In the presented study, co-expression of all xylose metabolism pathway genes was abandoned. The lack of overexpression of xylose reductase, the enzyme directly responsible for the conversion of xylose to xylitol, reduces the level of the by-product. In the conducted cultures, xylitol was not detected based on the HPLC analysis. Strain AJD/XYL/ALE is characterized by a satisfactory growth rate, short lag phase and high biomass production, after fermentation on pure xylose based medium. The results obtained are comparable to other xylose utilization processes with a total use of 20 g/L xylose in 5 days or OD600 = 25.8 after 4 days of fermentation compared to OD600 = 26.8 obtained by *Y. lipolytica* Y14 strain with multiple genome modification [38]. An essential part of the study was the production of lipids on waste substrate as a carbon source, rye straw hydrolysate. The total biomass productivity of AJD/XYL/ALE on hydrolysate was almost 2.5 times higher than reached during growth on pure xylose (46 mg/L/h compared to 18.06 mg/L/h) and 1.6 times higher than wild *Y. lipolytica* A101 growth on lignocellulosic hydrolysate (28 mg/L/h). The obtained strain showed enhanced synthesis of SCO without any major delay in growth. Both strains AJD/XDH/XK and AJD/XYL/ALE showed the same level of lipid accumulation above 35%. The improved availability of xylose in production medium for AJD/XYL/ALE strain resulted in an increased biomass levels. The process based on hydrolysate from agave bagasse and ylXYL + Obese strain allowed to obtain over 10 g/L biomass with 24.5% lipid content [46]. The shake-flask experiment showed that rye straw hydrolysate is a proper low-cost medium for fatty acid bioproduction. A number of valuable studies on lipid production by *Y. lipolytica* have been carried out to date. Higher level of lipid content (grams of lipid per gram of cell dry weight) have been obtained in bioprocess using glucose as substrate—in the range of 77–80% [54–56]. However, glucose-based processes generate high production costs. To reduce the outlay on lipid production, alternative carbon sources, such as glycerol, have begun to be used. Glycerol as a carbon source made it possible to obtain a wide range of lipid content. On glycerol as a sole substrate 52% lipid content was obtained [35], a combination of glycerol and molasses allowed for synthesis of 31% lipids [57], and glycerol enriched with xylose enabled the production of 42% lipids [44]. However, not all lignocellulosic raw materials are mainly composed of xylose. In rice bran hydrolysate, glucose was the dominant sugar [58]. The lipid content of *Y. lipolytica* was over 48%. Due to complex and differentiated chemical composition of lignocellulosic materials, they give the possibility to select the most appropriate substrate according to the purpose of bioproduction. In addition to the amount of lipids accumulated in cells, the fatty acids profile is also influential. It is worth noting that overexpression of *DGA1* resulted in a slightly different fatty acid pool in the biomass. An overall increase in saturated fatty acids (palmitic acid C16:0, stearic acid C18:0, behenic acid C22:0 and lignoceric acid C24:0) was observed. This is in agreement with the previous reports [1]. During the shake-flask experiment, the major fatty acid in the pool was oleic acid C18:1—partial *Y. lipolytica* A101 contained 50%, AJD/XYL/ALE increased this amount to over 63%. The domination of the acid C18:1 is typical for *Y. lipolytica* biolipids [59]. Interestingly, over half the linoleic acid (and isomers of C18:2 linolelaidic acid) content was reduced in xylose-targeted modified yeast strains. Prior researchers have demonstrated that various types of the provided substrate can affect the fatty acid profile [60]. The genetic modification of yeast also appears to have an effect on the lipid pool [1]. The present experiment verified that the medium based on a lignocellulosic waste such as rye straw is a relevant, low-cost medium for microbial lipid production by *Y. lipolytica*. The results provide a promising starting point for optimization aimed at enhancing the SCO titer.

## Conclusions

This study showed that overexpression of the native xylitol dehydrogenase (XDH) and xylulose kinase (XK) genes in *Y. lipolytica* allowed utilization of xylose as a sole carbon source. Subsequently, the prolonged-term shake-flask culture was used to reduce the duration of the lag phase and increase biomass level. The study carried out on a yeast strain that was previously prepared to overproduce lipid enabled the efficient conversion of xylose into microbial lipids. Rye straw hydrolysate, mainly composed of xylose and glucose, was thereafter tested as a carbon source for single cell oil production. The most efficient growth was observed with the strain AJD/XYL/ALE, and the lipid titer was improved tenfold over the control. This study provides evidence of low-cost bioproduction of lipids from lignocellulosic material. It also provides prospects for process optimization studies. The presented results brought to development one of the elements of green-biotechnology processes for enabling industrial waste management.

## Methods

### Strains and plasmids

Primary strains used in this study were *Y. lipolytica* A101 [61] and AJD with overexpression of *YALI0E32769g*—diacylglycerol acyltransferase Dga1p [40]. Both strains belong to the Department of Biotechnology and Food Microbiology at Wroclaw University of Environmental and Life Sciences, Poland. *Escherichia coli* DH5α was primarily used for molecular cloning. Vector pAD [62], carrying the UAS1_B16_-TEF promoter, was the basis for developing new plasmids with the native XR, XDH or XK gene fragment. All plasmids and strains used in this study are listed in Additional file 1: Table S1 in the supplemental material. The all XYL genes fragments were amplified from the *Y. lipolytica* A101 genomic DNA. The list of primers used is shown in Additional file 1: Table S2. The PCR amplified genes were cloned into the pAD vector using SgsI and NheI/Pml1 sites, T4 DNA Ligase (Thermo Fisher Scientific) and used for transformation of *E. coli*. The obtained plasmids were isolated using the Plasmid Mini Kit (A&A Biotechnology, Poland), sequenced (Genomed, Poland) and digested with MssI. Linear expression cassettes were used to transform yeast according to the lithium acetate method [63]. The restriction enzymes were acquired from FastDigest Thermo Scientific.

### Culture media

For molecular cloning in *E. coli* LB medium (A&A Biotechnology, Poland) was used supplemented with 100 µg/mL ampicillin for selection after transformation. For the yeast inoculum preparation 6.7 g/L YNB (Yeast Nitrogen Base, Merck, Germany) supplemented by 20 g/L of glucose was used.

(i) **Pure xylose-based medium** Growth of *Y. lipolytica* was conducted at 28 °C, 250 rpm in a 300 mL flask. The flask contained 50 mL of medium consisting of 20 g/L xylose (Sigma, Germany) and 6.7 g/L YNB. The same medium was used during growth tests.
(ii) **Transcript quantification** For RNA isolation the strains were grown for 24 h in medium consisting of 6.7 g/L YNB (Yeast Nitrogen Base, Merck, Germany) and 20 g/L glucose (Merck, Germany).
(iii) **Agricultural waste medium** *Y. lipolytica* A101, AJD-D/XDH/XK and AJD-D/XYL/ALE were used for biomass and lipid production with waste-based medium. 50 mL of rye straw hydrolysates [22] was supplemented with 6.7 g/L YNB (Yeast Nitrogen Base, Merck, Germany), sterilized by membrane filtration (Stericup Filter Units, 0.22 µm Durapore, Merck Millipore) and used for growth test and shake-flask experiments.

### Growth cultures

The AJD-D/XDH/XK strain was selected for cultivation aimed at reducing phase lag. The process lasted for 80 days. Every 7 days the current culture standardized to OD600 = 0.01 was used as an inoculum for the next fresh medium. The composition of the medium remained constant for each passages. Another 1 mL of current culture was immediately frozen with 0.7 mL of 30% glycerol in liquid nitrogen and stored at − 80 °C. Quantification of xylose was executed every day. The strain obtained after the last passage was named AJD-D/XYL/ALE. Then, the growth of the wild and all newly developed strains were tested in the Spark microplate reader (Tecan Group Ltd., Switzerland). The overnight inoculation cultures were centrifuged, washed with sterile water and standardized to OD600 = 0.15. The strains were grown in 100-well plates in 200 μL of xylose-based medium or waste-based medium. Quadruple experiments were performed under a constant agitation rate at 28 °C with measurement of optical density at 420–560 nm every 30 min for 72 h. Negative controls with no yeast were included.

### RNA isolation and transcript quantification

For RNA isolation cultures after 24 h of growth were centrifuged for 5 min at 12,000×*g*. Biomass was treated using the Total RNA Mini Plus kit (A&A Biotechnology, Poland). The obtained RNA was standardized to an equal concentration and additionally purified by DNase I (Thermo Scientific, USA) according to the producer’s instructions. Quantification of RNA was executed using a Biochrom WPA Biowave II spectrophotometer (Biochrom Ltd., UK) equipped with a TrayCell (Hellma Analytics, Germany). Maxima First Strand cDNA Synthesis kits for RT-qPCR (Thermo Fisher Scientific) were used for cDNA synthesis. For qRT-PCR analyses the DyNAmo Flash SYBR Green qPCR Kit (Thermo Fisher Scientific) was applied with the Eco Real-Time PCR System (Illumina, USA). The list of primers used is shown in Additional file 1: Table S2. Each sample was evaluated in triplicate. Expression level of genes was normalized to the actin gene. Data were analyzed using the ddCT method [64].

### Analytical method

The growth curves were determined by monitoring at 24-h intervals the optical density (OD_600_) with a UV spectrometer (SmartSpec Plus, Bio-Rad, USA) with a semi-micro cuvette (Sarstedt, Germany). Quantitative analysis of biomass was determined gravimetrically after centrifuging (5 min; 5500×*g*) 10 mL of samples from the shake-flask cultures, washed with distilled water, harvested by filtration on 0.22-μm membranes and drying at 105 °C.

(i) **HPLC** The concentrations of xylose were determined by HPLC using a Carbohydrate Analysis Column Aminex HPX-87P (Bio-Rad, USA) coupled to a UV (Dionex, USA) and a refractive index (RI) detector Shodex (Showa Denko K.K, Japan). The column was eluted with sterile water at 65 °C and a flow rate of 0.6 mL/min.
(ii) **GC–MS** Lipids from 15 to 20 mg of lyophilized biomass were converted into their methyl esters according the method described before [65]. Fatty acids were identified and quantified by comparison to reference material (37 FAME MIX, CRM47885, Sigma-Aldrich). GC–MS analysis was performed with a Shimadzu single quadrupole GCMS-QP2010SE instrument equipped with a Zebron ZB-FAME column. Hexane was used as a solvent, and helium was used as a carrier gas (linear velocity—35 cm/s).

### Calculation of fermentation parameters

The formula Yc = X/S was used to calculated the biomass total yield. X mean the final amount of biomass after at the end of the experiment. S mean the total amount of sugar in the hydrolysate. The total lipid yield was calculated in in the same manner.

The formula Qx = X/120 was used to calculated the biomass total productivity. An analogical formula was use to calculated the total lipid productivity.

The formula Qs = ΔS/144 was used to calculated the xylose consumption rate.

### Statistical analysis

Statistical analysis of gene expression data, one-way ANOVA, were conducted using Statistica 12.5 (StatSoft, Kraków, Poland). Significant differences (p ≤ 0.05) between mean were assessed by Duncan’s t-test.

## Supplementary Information

**Additional file 1: Figure S1.** Schematic for xylose utilization pathway and new *Y. lipolytica* strains preparation. **Table S1.** Strains and plasmids used in presented study. **Table S2.** List of primers.

## Data Availability

Not applicable.
