# Supplementary material for: Lipid production from lignocellulosic biomass using an engineered Yarrowia lipolytica strain
Source: Microb Cell Fact. 2022 Oct 28;21:226. doi: 10.1186/s12934-022-01951-w (PMC9617373; doi:10.1186/s12934-022-01951-w)
Supplement: Supplementary file 1 — Additional file 1: Figure S1. Schematic for xylose utilization pathway and new Y. lipolytica strains preparation. Table S1. Strains and plasmids used in presented study. Table S2. List of primers. [file 12934_2022_1951_MOESM1_ESM.docx]

**Additional file 1**

**Lipid production from lignocellulosic biomass using an engineered and adapted *Yarrowia lipolytica* strain**

Katarzyna Drzymała-Kapinos^[[1]](#footnote-1)^, Aleksandra M. Mirończuk1_,_ Adam Dobrowolski1*

^1^Department of Biotechnology and Food Microbiology, Wrocław University of Environmental and Life Sciences, Wrocław, Poland

*Corresponding Author – Email: adam.dobrowolski@upwr.edu.pl Address: University of Environmental and Life Sciences, Department of Biotechnology and Food Microbiology, 37 Chełmońskiego Street 51-630 Wrocław, Poland

Figure S1. Schematic for xylose utilization pathway and new *Y. lipolytica* strains preparation.


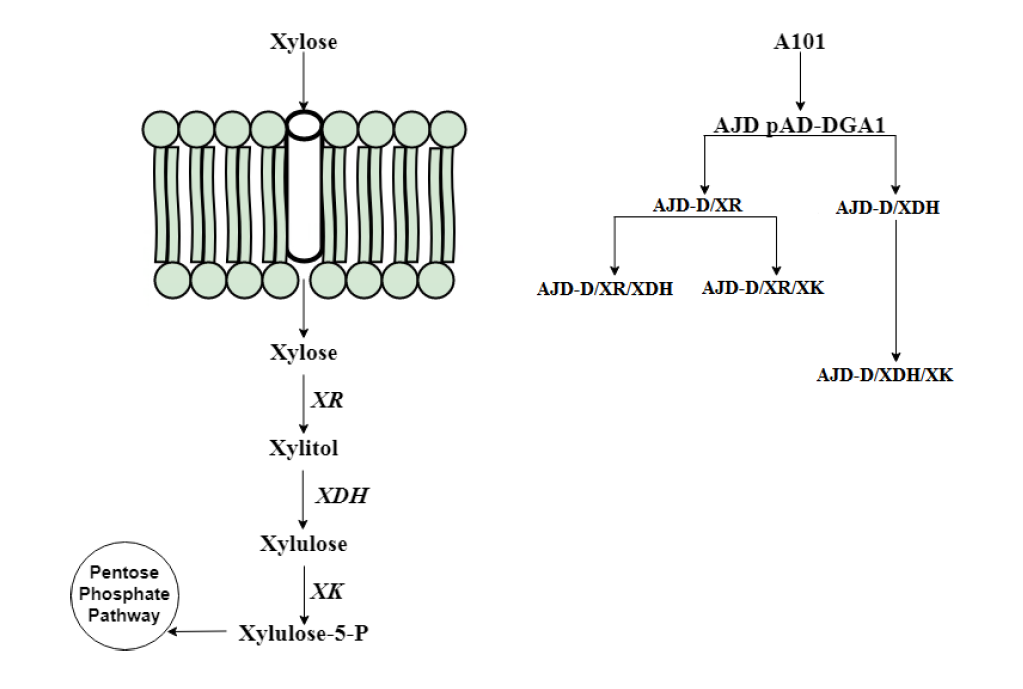


Table S1. Strains and plasmids used in presented study

| **Strain** | **Genotype or plasmid** | **Source** |
| --- | --- | --- |
| *E. coli* |  |  |
| DH5α | F− endA1 glnV44 thi-1 recA1 relA1 gyrA96 deoR nupG  Φ80dlacZΔM15 Δ(lacZYA-argF)U169, hsdR17(rK-mK+), λ− | Hanahan D. DNA cloning: a practical approach. In: Glover DM, editor.  McLean: IRL Press; 1985. |
|  |  |  |
| DH5α | pAD-XR, *YALI0D07634g* | This study |
| DH5α | pAD-XDH, *YALI0E12463g* | This study |
| DH5α | pAD-XYL3, *YALI0F10923g* | This study |
| *Y. lipolytica* |  |  |
| A101 | Wild type | Wojtatowicz et al., 1991 |
| AJD | *MATA,* A101: ura3-302 | Mironczuk et al., 2015 |
| AJD-D | *MATA,* A101: ura3-302 overexpression *YALI0E32769g* | This study |
| AJD-D/XR/XDH | *MATA,* A101: ura3-302 overexpression *YALI0E32769g , YALI0D07634g* and *YALI0E12463g* | This study |
| AJD-D/XR/3 | *MATA,* A101: ura3-302 overexpression *YALI0E32769g , YALI0D07634g* and *YALI0F10923g* | This study |
| AJD-D/XHD/3 | *MATA,* A101: ura3-302 overexpression *YALI0E32769g , YALI0E12463g* and *YALI0F10923g* | This study |
| AJD-D/XYL/ALE | *MATA,* A101: ura3-302 overexpression *YALI0E32769g , YALI0E12463g* and *YALI0F10923g*, with a short lag phase | This study |

Table S2. List of primers

| **Gene** | **Plasmid** | **Sequence** |
| --- | --- | --- |
| *Primers used for constructing plasmids* | |  |
| *YALI0D07634g* | XR_Asc1_F | ACGGGCGCGCCATGTCCTTCAAGCTCGCCTCCGGAAAG |
|  | XR_Nhe1_R | AGCGCTAGCTCCGTGTTTCCACGCTTAGG |
| *YALI0E12463g* | XDH_Asc1_F | ACGGGCGCGCCATGTCTTCTAACCCGTCATTTG |
|  | XDH_Pml1_R | CACGTGCCGGTTGTAGATTCACTAAG |
| *YALI0F10923g* | XK_Asc1_F | ATGGGCGCGCCATGTATCTCGGACTGGATCTTTCG |
|  | XK_Nhe1_R | ACGGCTAGCGCCATGCTGGTTTAGTTTAC |
| *Primers used for RT-PCR* | |  |
| *YALI0D07634g* | q_XR_F | GATTCGGCCTGTGGAAGGTC |
|  | q_XR_R | TTGATGGCTCGTCGGATACC |
| *YALI0E12463g* | q_XDH_F | GCCATTGGATCTCGTCTTTG |
|  | q_XDH_R | CCTCCATGCAGATAGTAGTG |
| *YALI0F10923g* | q_XK_F | CGGACTGGATCTTTCGACTC |
|  | q_XK_R | CCACTGTAGAGCTGTGAAAG |
| *YALI0E32769g* | q_DGA1_F | TCGCGGGAATCCGATATGCC |
|  | q_DGA1_R | GTGGAATTGCGCAGCATAGC |
| *actin* | q_ACT_F | GAGTCACCGGTATCGTTC |
|  | q_ACT_R | GCGGAGTTGGTGAAAGAG |

1. *Corresponding author: Adam Dobrowolski, University of Environmental and Life Sciences, Department of Biotechnology and Food Microbiology, 37 Chełmońskiego Street 51-630 Wrocław, Poland, Tel: 71 3207791, e-mail: adam.dobrowolski@upwr.edu.pl [↑](#footnote-ref-1)
